# Increasing mean arterial blood pressure in sepsis: effects on fluid balance, vasopressor load and renal function

**DOI:** 10.1186/cc12495

**Published:** 2013-01-30

**Authors:** Thiago Domingos Corrêa, Madhusudanarao Vuda, Jukka Takala, Siamak Djafarzadeh, Eliézer Silva, Stephan Mathias Jakob

**Affiliations:** 1 Department of Intensive Care Medicine, Inselspital, Bern University Hospital and University of Bern, Freiburgstrasse 10, Bern, CH-3010, Switzerland; 2 Intensive Care Unit, Hospital Israelita Albert Einstein, Av Albert Einstein 627/701, São Paulo, 05651-901, Brazil

## Abstract

**Introduction:**

The objective of this study was to evaluate the effects of two different mean arterial blood pressure (MAP) targets on needs for resuscitation, organ dysfunction, mitochondrial respiration and inflammatory response in a long-term model of fecal peritonitis.

**Methods:**

Twenty-four anesthetized and mechanically ventilated pigs were randomly assigned (n = 8/group) to a septic control group (septic-CG) without resuscitation until death or one of two groups with resuscitation performed after 12 hours of untreated sepsis for 48 hours, targeting MAP 50-60 mmHg (low-MAP) or 75-85 mmHg (high-MAP).

**Results:**

MAP at the end of resuscitation was 56 ± 13 mmHg (mean ± SD) and 76 ± 17 mmHg respectively, for low-MAP and high-MAP groups. One animal each in high- and low-MAP groups, and all animals in septic-CG died (median survival time: 21.8 hours, inter-quartile range: 16.3-27.5 hours). Norepinephrine was administered to all animals of the high-MAP group (0.38 (0.21-0.56) mcg/kg/min), and to three animals of the low-MAP group (0.00 (0.00-0.25) mcg/kg/min; *P *= 0.009). The high-MAP group had a more positive fluid balance (3.3 ± 1.0 mL/kg/h vs. 2.3 ± 0.7 mL/kg/h; *P *= 0.001). Inflammatory markers, skeletal muscle ATP content and hemodynamics other than MAP did not differ between low- and high-MAP groups. The incidence of acute kidney injury (AKI) after 12 hours of untreated sepsis was, respectively for low- and high-MAP groups, 50% (4/8) and 38% (3/8), and in the end of the study 57% (4/7) and 0% (*P *= 0.026). In septic-CG, maximal isolated skeletal muscle mitochondrial Complex I, State 3 respiration increased from 1357 ± 149 pmol/s/mg to 1822 ± 385 pmol/s/mg, (*P *= 0.020). In high- and low-MAP groups, permeabilized skeletal muscle fibers Complex IV-state 3 respiration increased during resuscitation (*P *= 0.003).

**Conclusions:**

The MAP targets during resuscitation did not alter the inflammatory response, nor affected skeletal muscle ATP content and mitochondrial respiration. While targeting a lower MAP was associated with increased incidence of AKI, targeting a higher MAP resulted in increased net positive fluid balance and vasopressor load during resuscitation. The long-term effects of different MAP targets need to be evaluated in further studies.

## Introduction

The arterial blood pressure is the driving force to blood flow through the tissues. According to the principle of blood flow autoregulation, if cardiac output is constant, blood flow to tissues does not change until blood pressure falls below a critical value. When this autoregulatory threshold is reached, any additional decrease in arterial blood pressure levels will compromise organ blood flow [1].

The Surviving Sepsis Campaign Guidelines recommend a mean arterial blood pressure (MAP) higher than 65 mmHg during the initial resuscitation of severe sepsis and septic shock [2]. However, there is only weak evidence to support this recommendation.

Few small prospective clinical studies have addressed the effects of different MAP levels on tissue perfusion in septic shock [3-6]. Increasing MAP from 65 to 85 mmHg has not been shown to improve systemic oxygen metabolism, splanchnic perfusion or renal function, has had inconsistent effects on local microcirculation, and has been associated with higher left ventricular stroke work index and increased exposure to norepinephrine [3,4,6]. Apart from influencing systemic and regional perfusion and organ blood flow distribution [7,8], vasopressors may also affect the balance between oxygen and substrate supply and metabolic needs [9], with the potential to affect mitochondrial function [10], which has been associated with organ failure and poor outcome during sepsis [11-13]. Furthermore, in clinical practice as well as in research, the actual blood pressure achieved is often markedly higher than the targets originally prescribed [14], resulting in unnecessary and potentially harmful, further exposure to fluids and catecholamines [15].

The optimal balance between arterial blood pressure goals and minimal side-effects of fluids and drugs needs to be determined [16]. On one hand, aiming for higher MAP levels during the initial resuscitation of patients in septic shock increases the exposure to fluids, vasopressors and inotropes, which has been associated with increased mortality [15]. On the other hand, targeting lower levels of MAP may increase the incidence of tissue hypoperfusion and contribute to progression of organ failure [17-19].

The aim of this experimental study was to evaluate the impact of two different levels of MAP, namely between 50 and 60 mmHg (low-MAP) or between 75 and 85 mmHg (high-MAP), on the need for resuscitation, sepsis-associated organ dysfunction, mitochondrial respiration and inflammatory response in a long-term model of fecal peritonitis.

## Materials and methods

This study was performed in accordance with the National Institutes of Health guidelines for the care and use of experimental animals and with the approval of the Animal Care Committee of the Canton Bern, Switzerland.

Twenty-four domestic pigs of both sexes (mean weight ± SD 41.0 ± 3.1kg) were fasted for 12 hours prior to the experiment, with free access to water. The pigs were sedated with intramuscular ketamine (20 mg/kg) and xylazine (2 mg/kg). A peripheral intravenous catheter was inserted in an ear vein for administration of fluids and drugs. Pigs were orally intubated following induction of anesthesia with midazolam (0.5 mg/kg) and atropine (0.02 mg/kg). Anesthesia was maintained with propofol (4 mg/kg/h) and fentanyl (5 µg/kg/h during surgery and 2 µg/kg/h afterwards). When necessary, additional injections of fentanyl (50 µg) or midazolam (5 mg) were administered. The stomach was kept empty by insertion of a large-bore orogastric tube.

Animals were ventilated in a volume-controlled mode with a positive end-expiratory pressure (PEEP) of 5 cm H_2_O, a fractional inspiratory oxygen concentration (FiO_2_) of 30%, and a tidal volume of 8 mL/kg (Servo-i^®^, Maquet Critical Care, Solna, Sweden). The respiratory rate was adjusted aiming an arterial carbon dioxide partial pressure (PaCO_2_) between 35 and 45 mmHg.

### Surgical preparation

The right jugular vein was cannulated via a midline cervical incision for a pulmonary artery catheter placement. A double lumen catheter was placed into the left femoral vein. An arterial catheter for blood pressure measurement and blood sampling was placed into the right carotid artery. Ultrasound Doppler 4-mm flow probes (Transonic^® ^Systems Inc., Ithaca, NY, USA), which had previously been calibrated *in vitro*, were placed around the left carotid artery and the left femoral artery. With the pig in the supine position, a midline mini-laparotomy was performed and a large-bore intra-peritoneal drain inserted. After preparation, the abdominal incision was closed. Finally, cystostomy was performed for drainage of urine. Throughout the surgical preparation, Ringer's lactate was infused at 3 mL/kg/h.

### Study protocol

**Figure 1:**
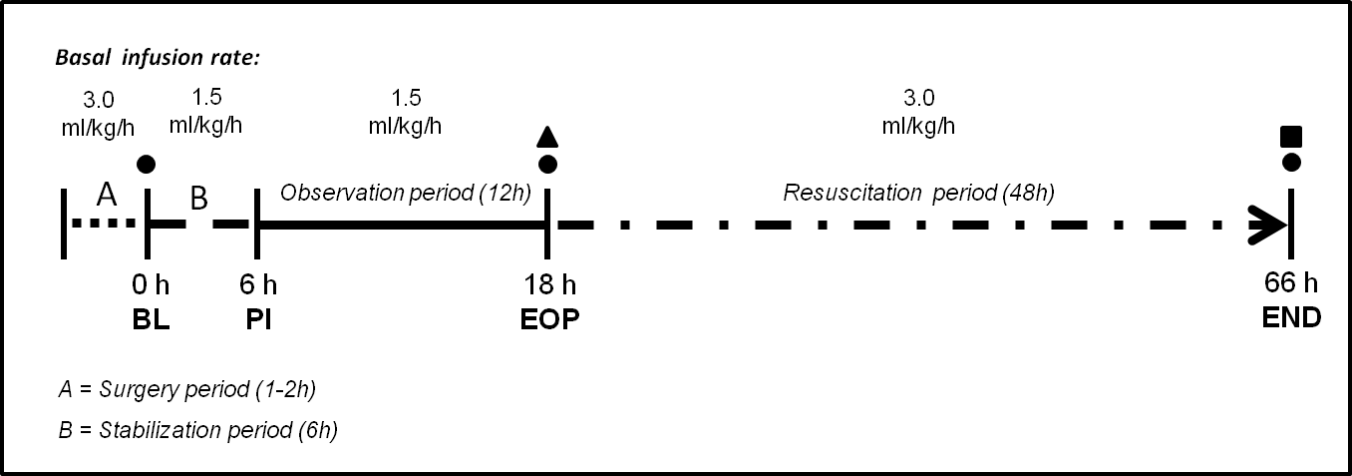
**Study design for the low and high mean arterial pressure (MAP) groups**. Black circles, samples from the right quadriceps muscle to assess mitochondrial function and quantification of skeletal muscle ATP content; black triangle, first dose of intravenous antibiotic (piperacillin/tazobactam, 2.25 g, 8-hourly); black square, additional tissue samples from the liver for mitochondrial function analysis at the end of the experiment (66 h or before death, if earlier). Animals allocated to the septic control group (Septic-CG) were followed without resuscitation after induction of peritonitis until death. BL, baseline; PI, peritonitis induction; EOP, end of observation period (before starting resuscitation); End, end of the experiment (at 48 hours of resuscitation or before death if earlier.

After surgical preparation, animals were stabilized for 6 h and Ringer's lactate and glucose 50% (G50%) were infused (adding up to 1.5 mL/kg/h) and adjusted to keep blood glucose in the range of 3.5 to 5.0 mmol/L. Then, baseline measurements were performed and pigs were randomly assigned (n = 8 per group) to a septic control group (septic-CG) without resuscitation until death, or to one of two groups in which resuscitation was performed after 12 h of untreated sepsis for 48 h, targeting MAP between 50 and 60 mmHg (low-MAP) or between 75 and 85 mmHg (high-MAP) (Figure 1). Fecal peritonitis was induced by peritoneal instillation of 2 g/kg body weight of autologous feces dissolved in 250 mL warmed glucose 5% solution. Afterwards, the intra-peritoneal drain remained clamped throughout the experiment.

After 12 h of untreated sepsis (observation period), animals randomized to the low- and high-MAP groups were submitted to 48 h of protocolized resuscitation (resuscitation period) conducted by trained intensivists (Additional file 1). Alternating boluses of 150 mL Ringer's lactate and 6% hydroxyethyl starch (130/0.4) were administered when signs of hypovolemia became evident. Fluid boluses were repeated as long as the stroke volume was increased by 10% or more after fluid administration. The maximum dose of hydroxyethyl starch administered during the study was 30 mL/kg. After this maximal dose was reached, only boluses of Ringer's lactate were given. If the mixed venous oxygen saturation (SvO_2_) was lower than 50%, dobutamine administration was started at a dose of 5.0 mg/h. This dose was increased by 5.0 mg/h every 30 minutes until the SvO_2 _was 50% or higher, or until a maximal dose of 20 mg/h was given. If the MAP was below 50 mmHg in the low-MAP group and 75 mmHg in the high-MAP group, norepinephrine was administrated (Figure S1 in Additional file 2).

After resuscitation was started, all animals in the low- and high-MAP groups received intravenous (i.v.) administration of piperacillin-tazobactam (Tazobac^®^) 2.25 g, every 8 h, and deep vein thrombosis prophylaxis (continuous i.v. infusion of 10,000 IU of non-fractionated heparin/24 h) (Figure 1). Details on respiratory care and ventilator settings can be found in Additional file 1. Following 48 h of resuscitation, study animals were deeply sedated and euthanized with an overdose of potassium chloride.

A basal fluid supply of 1.5 mL/kg/h during the observation period and 3.0 mL/kg/h during the resuscitation period was given as Ringer's lactate and G50%, adjusted to maintain blood glucose in the range of 3.5 to 5.0 mmol/L.

### Monitoring

Hemodynamics, temperature and respiratory parameters were monitored throughout the study, using a multi-modular patient monitor (S/5 Critical Care Monitor^®^; Datex-Ohmeda, GE Healthcare, Helsinki, Finland). Thermodilution cardiac output (L/minute), mixed venous oxygen saturation (SvO_2_) (Vigilance^®^; Edwards Lifesciences LLC, Irvine, CA, USA), and carotid and femoral artery blood flows (TS 420 double-channel flowmeters, Transonic Systems Inc.) were continuously measured. Data were recorded in software for data acquisition and signal analysis (Soleasy™, National Instruments Corp., Austin, TX, USA), and in an electronic patient data management system (Centricity Clinisoft^®^; GE Healthcare).

### Blood sampling

Techniques and time points of blood sampling for determination of blood gases, arterial lactate, electrolytes, hemoglobin, platelets, white blood cell count, serum creatinine, total bilirubin, creatine-kinase, troponin, plasma IL-6 andTNF-α are described in Additional file 1.

### Mitochondrial function analysis

Right quadriceps muscle samples were taken to assess isolated skeletal muscle, mitochondrial function of permeabilized skeletal muscle fibers, and for the quantification of skeletal muscle, ATP content. Isolation of skeletal muscle, liver mitochondria and mitochondrial oxygen consumption (by high-resolution respirometry) were performed as described previously [20]. An additional tissue sample was taken from the left lobe of the liver at the end of the experiment (Figure 1). Preparation of permeabilized muscle fibers is described in detail in Additional file 1.

In septic-CG, respiration of isolated skeletal muscle mitochondria and permeabilized skeletal muscle fibers was assessed at baseline, and before death occurred (end). In the low- and high-MAP groups, isolated skeletal muscle mitochondria and permeabilized skeletal muscle fibers mitochondrial respiration were measured at baseline, end of the observation period (EOP; before starting resuscitation) and at the end of the experiment (END). In animals that died earlier, the final samples were taken when the animals were still alive and receiving the maximal norepinephrine dose of 5,000 mcg/h, and when MAP approached 30 mmHg. Skeletal muscle ATP content was quantified using a luciferase-based ATP determination kit (A22066, Molecular Probes, Eugene, OR, USA).

### Calculations and acute kidney injury definition

Systemic oxygen delivery (DO_2_; mL/min) and consumption (VO_2_; mL/min) were calculated using cardiac output and arterial and mixed venous blood gases, according to standard formulas (Additional file 1). Acute kidney injury (AKI) was defined according to the AKIN criteria as an increase of more than 0.3 mg/dl (26.4 μmol/L) or 150% in serum creatinine from the baseline measurement [21]. Percentage of time below or above a certain blood pressure was calculated using 2-minute median-filtered values.

### Statistical analysis

All data are presented as mean ± standard deviation (SD), or median with IQR in the case of non-normal distribution (tested by the Kolmogorov-Smirnov test). All baseline data, fluids, vasoactive drugs, sedation, fluid balance, creatine-kinase, troponin, liver mitochondrial respiration and skeletal muscle ATP content were compared between groups using one-way analysis of variance (ANOVA) followed by the Tukey or independent samples *t*-test. Binary variables were analyzed with Fisher's exact test and survival proportions between groups were analyzed with the log rank test.

Differences between groups in the evolution over time were assessed by ANOVA for repeated measurements using group as the between-subject factor and time as the within-subject factor. This was done first for all three groups, using baseline, EOP and end values. Afterwards, the same procedure was repeated for the low- and high-MAP groups only, but including all measurements (baseline, EOP, 12, 24, 36 and 48 h). If a time-group interaction was detected between the low- and high-MAP groups, the independent samples *t*-test was performed at the end of the experiment (at 48 h of resuscitation, or before death if earlier). In the case of non-normal distribution of study variables, time effect was separately measured within each group using the non-parametric Friedman's test. At the EOP and at the end of the experiment, all study groups were compared with Kruskal-Wallis test, and the low- and high-MAP groups were compared with Mann-Whitney *U*-test, in the case of a significant Kruskal-Wallis test. A potential relationship between time spent below/above different blood pressure levels expressed as percent, or being administered fluids, and end plasma creatinine concentrations/diuresis was evaluated using Spearman's correlation. The paired *t*-test was performed to compare baseline to end in the septic-CG.

A *P*-value <0.05 was considered significant. The Statistical Package for Social Sciences (SPSS) version 20.0 (SPSS Inc.^®^; Chicago, IL, USA) was used for statistical analysis.

## Results

### Mortality

**Figure 2:**
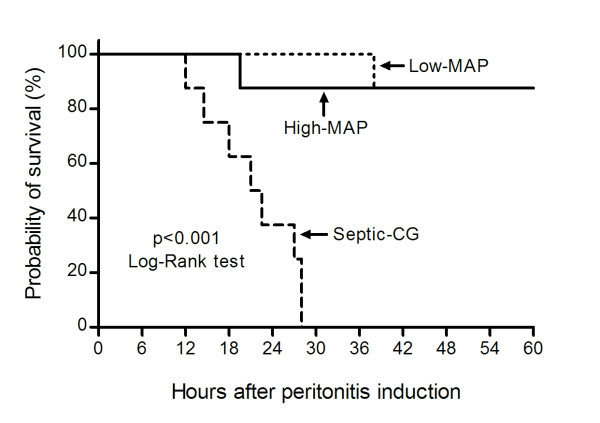
**Kaplan-Meyer survival curve**. MAP, mean arterial pressure; Septic-CG, septic control group.

All animals in the septic-CG died, with a median survival time of 21.8 hs (16.3 to 27.5 h). One animal each in the low- and high-MAP groups died during the resuscitation period, respectively, after 38 and 19.5 h of induction of peritonitis (Figure 2).

### Treatment intensity

The high-MAP group tended to receive more fluid boluses than the low-MAP group (*P *= 0.06) and had higher cumulative fluid balance at study end (*P *= 0.001) (Table 1). During the resuscitation period, norepinephrine was administered to 3/8 animals (37.5%) in the low-MAP group and to all animals in the high-MAP Group (*P *= 0.026) (Table 1; see also Figure S1 in Additional file 2). Dobutamine was administered to 4/8 animals (50.0%) in the low-MAP and to 3/8 animals (37.5%) in the high-MAP group (*P *= 1.0). Doses of administered norepinephrine and dobutamine are shown in Table 1.

**Table 1:** Administered treatment, fluid output and balance during the study

Variables/Study group	Septic-CG	Low-MAP	High-MAP	*P*-value
Propofol, mg/kg/h	5.0 ± 1.0	5.8 ± 0.7	5.8 ± 0.5	0.11^a^
Fentanyl, mcg/kg/h^†^	4.0 ± 1.6	3.9 ± 0.8	4.4 ± 0.9	0.62^a^
Midazolam, mg/kg/h	0.08 ± 0.03	0.06 ± 0.02	0.07 ± 0.04	0.38^a^
Fluid bolus, mL/kg/h^††*^				
Median		0.7	2.0	0.06^b^
IQR		0.5, 2.0	1.6, 2.8	
Norepinephrine				
Number/number in group (%)		3/8 (37.5)	8/8 (100.0)	0.026^c^
Median dose, mcg/kg/min		0.00	0.38	0.009^b^
IQR		0.00, 0.25	0.21, 0.56	
Dobutamine				
Number/number in group (%)		4/8 (50.0)	3/8 (37.5)	1.00^c^
Median dose, mcg/kg/min		0.06	0.00	0.45^b^
IQR		0.00, 3.16	0.00, 1.00	
Total volume received, mL/kg/h	1.5 ± 0.0	3.6 ± 0.8^§^	4.7 ± 1.4^#^	0.001^a^
Urine output, mL/kg/h	0.6 ± 0.1	1.0 ± 0.2^§^	1.1 ± 0.2^#^	0.001^a^
Gastric tube output, mL/kg/h	0.3 ± 0.2	0.3 ± 0.3	0.3 ± 0.2	0.95^a^
Final balance^╫^, mL/kg/h	0.6 ± 0.3	2.3 ± 0.7^§^	3.3 ± 1.0^#¥^	0.001^a^
Final balance^╫^, L	0.5 ± 0.4	5.2 ± 1.1^§^	6.7 ± 1.4^#¥^	0.001^a^

Values are mean ± SD or median (IQR) unless stated otherwise. ^†^Sum of basic fentanyl infusion with additional boluses; *administered during the 48-h resuscitation period; ^††^sum of Ringer's lactate and hydroxyethyl starch; ^╫^final balance = (Ringer's Lactate basal infusion + glucose 50% basal infusion + Ringer's lactate bolus + hydroxyethyl starch bolus) - (urine output + gastric tube output); ^a^one-way analysis of variance (ANOVA). Tukey post hoc test after ANOVA, comparisons significant at the 0.05 level: ^¥^low-mean arterial pressure (MAP) versus high-MAP; ^§^low-MAP versus septic control group (Septic-CG); ^#^high-MAP versus Septic-CG; ^b^Mann-Whitney test; ^c^Fisher's exact test.

### Hemodynamics, oxygen transport, respiratory and acid-base variables

MAP at the end of the resuscitation period was 56 ± 13 mmHg and 76 ± 17 mmHg respectively, for the low- and high-MAP groups (Table 2). During the resuscitation period, blood pressure attained overlapped to a certain extent between the groups: in the low-MAP animals it was ≥75 mmHg during 27.5% of the time, while it was <75 mmHg in the high-MAP group during 8.1% of the time (Table S1 in Additional file 2). None of the other hemodynamic parameters, systemic oxygen delivery, consumption, or arterial lactate differed between the low- and high-MAP groups (Table 2). Arterial pH, bicarbonate, base excess, PaCO_2_, minute ventilation, ratio of arterial oxygen partial pressure to fraction of inspired oxygen and PEEP did not differ between the low- and high-MAP groups (Table S2 in Additional file 2).

**Table 2:** Systemic and regional hemodynamics and arterial lactate level

Variable	Group	BL	EOP	RP 12 h	RP 24 h	RP 36 h	RP 48h	END	*P*-value^¶ ^
MAP,	Septic-CG	78 ± 9	68 ± 22					29 ± 4	<0.001**^a^**
mm Hg	Low-MAP	65 ± 9	81 ± 16	72 ± 13	67 ± 12	61 ± 6	59 ± 8	56 ± 13	<0.001**^a^**
	High-MAP	63 ± 7	76 ± 11	86 ± 5	81 ± 7	82 ± 4	82 ± 3	76 ± 17	
Heart rate,	Septic-CG	104 ± 17	193 ± 41					216 ± 25	<0.001^a^
beats/minute	Low-MAP	93 ± 11	166 ± 23	165 ± 14	156 ± 23	141 ± 28	122 ± 28	130 ± 46	0.84^b^
	High-MAP	105 ± 23	173 ± 31	163 ± 22	155 ± 31	138 ± 28	117 ± 29	117 ± 29	
MPAP,	Septic-CG	12 ± 2	16 ± 3					11 ± 2	<0.001**^a^**
mm Hg	Low-MAP	15 ± 3	18± 2	20 ± 5	21 ± 5	23 ± 6	23 ± 5	23 ± 4	0.19^b^
	High-MAP	13 ± 1	18 ± 3	19± 2	21 ± 1	24 ± 2	24 ± 3	27 ± 9	
CVP,	Septic-CG	1 ± 1	1 ± 1					0 ± 1	<0.001**^a^**
mm Hg	Low-MAP	4 ± 2	2 ± 2	3 ± 2	5 ± 3	7 ± 4	8 ± 4	9 ± 4	0.41^b^
	High-MAP	2 ± 1	1 ± 1	3 ± 2	6 ± 1	7 ± 1	9 ± 2	10 ± 3	
SVRI,	Septic-CG	692 ± 129	966 ± 197					600 ± 238	0.082**^a^**
mm Hg	Low-MAP	589 ± 60	923 ± 203	668 ± 131	451 ± 131	373 ± 56	349 ± 92	319 ± 119	0.19^b^
L/Kg/min	High-MAP	553 ± 87	1017 ± 304	698 ± 114	525 ± 185	488 ± 109	510 ± 107	475 ± 140	
Cardiac	Septic-CG	115 ± 27	70 ± 21					53 ± 19	<0.001**^a^**
index,	Low-MAP	105 ± 13	88 ± 22	104 ± 13	143 ± 31	145 ± 13	149 ± 19	150 ± 18	0.38^b^
mL/kg/min	High-MAP	112 ± 23	77 ± 16	122 ± 29	165 ± 78	160 ± 36	150 ± 31	142 ± 35	
SvO_2_,	Septic-CG	59 ± 8	53 ± 12					35 ± 11	0.001**^a^**
%	Low-MAP	48 ± 2	56 ± 9	58 ± 9	56 ± 9	60 ± 6	61 ± 7	55 ± 17	0.06**^b^**
	High-MAP	50 ± 8	51 ± 3	61 ± 6	66 ± 3	66 ± 5	67 ± 5	60 ± 20	
DO_2_,	Septic-CG	626 ± 163	592 ± 158					421 ± 113	<0.001**^a^**
mL/min	Low-MAP	526 ± 118	663 ± 172	682 ± 100	748 ± 99	712 ± 93	687 ± 149	687 ± 149	0.26^b^
	High-MAP	541 ± 92	566 ± 78	749 ± 118	919 ± 444	862 ± 201	744 ± 135	702 ± 173	
VO_2_,	Septic-CG	241 ± 27	267 ± 44					280 ± 91	0.293**^a^**
mL/min	Low-MAP	277 ± 46	284 ± 38	281 ± 28	298 ± 27	282 ± 35	254 ± 39	254 ± 39	0.95^b^
	High-MAP	269 ± 50	273 ± 44	280 ± 49	306 ± 120	276 ± 34	247 ± 30	256 ± 38	
CBFI,	Septic-CG	6.7 ± 1.3	3.9 ± 0.5					2.8 ± 0.6	<0.001**^a^**
mL/kg/min	Low-MAP	6.9 ± 2.2	4.9 ± 2.1	6.4 ± 1.9	8.5 ± 2.1	8.4 ± 1.6	8.0 ± 1.6	7.6 ± 1.8	0.55^b^
	High-MAP	5.4 ± 1.6	4.1 ± 1.2	5.6 ± 1.6	6.7 ± 1.6	6.8 ± 1.8	6.2 ± 1.8	5.8 ± 2.0	
FBFI,	Septic-CG	3.5 ± 0.7	1.7 ± 0.5					1.2 ± 0.3	<0.001**^a^**
mL/kg/min	Low-MAP	3.9 ± 1.6	1.7 ± 0.8	2.2 ± 0.4	3.4 ± 1.0	3.8 ± 0.6	3.9 ± 0.9	3.7 ± 1.0	0.33**^b^**
	High-MAP	3.1 ± 0.9	1.4 ± 0.6	2.2 ± 0.7	3.2 ± 1.0	3.2 ± 0.8	3.4 ± 0.7	3.1 ± 1.0	
Arterial	Septic-CG	0.7 ± 0.1	1.9 ± 1.2					4.1 ± 2.0	<0.001**^a^**
lactate,	Low-MAP	1.3 ± 0.6	1.0 ± 0.4	1.2 ± 0.3	1.1 ± 0.4	1.1 ± 0.5	0.9 ± 0.2	1.0 ± 0.4	0.19^b^
mmol/L	High-MAP	0.9 ± 0.3	1.0 ± 0.2	1.2 ± 0.5	1.2 ± 0.5	1.1 ± 0.2	1.1 ± 0.3	1.5 ± 1.4	

Values are mean ± SD. BL, baseline; EOP, end of observation period for the low-MAP and high-MAP groups (12 h after induction of peritonitis); RP, resuscitation period; END, end of the experiment (at 48 hours of resuscitation, or before death if earlier); MAP, mean arterial blood pressure; MPAP, mean pulmonary artery pressure; PAOP, pulmonary artery occlusion pressure; CVP, central venous pressure; SVRI, systemic vascular resistance index; SvO_2_, mixed venous oxygen saturation; DO_2, _systemic oxygen delivery; VO_2, _systemic oxygen consumption; CBFI, carotid artery flow index; FBFI, femoral artery flow index; ^¶^*P*-value for ^a^time-group interaction with repeated measures analysis of variance (ANOVA) including BL, EOP and END for all study groups (septic control (Septic-CG), Low-MAP and High-MAP groups); ^b^time-group interaction with repeated measures ANOVA including BL, EOP, RP 12 h, RP 24 h, RP 36 h and RP 48 h for the Low-MAP and High-MAP groups.

### Inflammatory markers and organ function parameters

IL-6 and TNF- α plasma levels increased from baseline to the EOP in both the low- and high-MAP groups, and decreased during the resuscitation period (Table 3). Platelets, white blood cell count, bands, metamyelocytes, total bilirubin, creatine-kinase and troponin did not differ between the low- and high-MAP groups at study end (Table 3). However, animals allocated to low-MAP exhibited a higher median plasma creatinine level than animals in the high-MAP group (*P *= 0.021) (Table 3; see also Figure S2 in Additional file 2). The incidence of AKI after 12 h of untreated sepsis was, respectively for the low- and high-MAP groups, 50% (4/8) and 38% (3/8) (*P *= 1.00). At the end of the study, 57% (4/7) in the low-MAP group and none in the high-MAP group had AKI (*P *= 0.026).

**Table 3:** Inflammatory markers and organ function analysis

Variable	Group	BL	EOP	End	*P*-value^¶ ^
IL-6,	Septic-CG	24 ± 44	781 ± 410*	1785 ± 819	<0.001**^a^**
pg/mL	Low-MAP	20 ± 26	291 ± 165	57 ± 59**^#^**	0.61**^b^**
	High-MAP	5 ± 10	258 ± 107	95 ± 177	

TNF-α,	Septic-CG	102 ± 75	290 ± 145*	410 ± 160	<0.001**^a^**
pg/mL	Low-MAP	48 ± 16	149 ± 55	93 ± 45**^#^**	0.52**^b^**
	High-MAP	57 ± 39	130 ± 89	76 ± 65	

Hemoglobin,	Septic-CG	9.4 ± 0.8	15.4 ± 1.6	14.7 ± 1.8	<0.001**^a^**
mg/dL	Low-MAP	9.3 ± 0.6	14.7 ± 0.7	8.4 ± 0.8	0.014**^b^**
	High-MAP	9.2 ± 0.4	14.8 ± 1.2	10.0 ± 1.2	0.008**^c^**

Platelets,	Septic-CG	296 ± 97	131 ± 66*	98 ± 53	0.18**^a^**
x 10^9^/L	Low-MAP	255 ± 48	176 ± 41	115 ± 42**^#^**	0.19**^b^**
	High-MAP	323 ± 102	131 ± 66	98 ± 53	

White blood cell count,	Septic-CG	14.0 ± 1.6	9.7 ± 3.0*	10.7 ± 2.8	0.013**^a^**
x 10^9^/L	Low-MAP	18.4 ± 5.2	12.6 ± 4.1	16.9 ± 5.2**^#^**	0.014**^b^**
	High-MAP	16.9 ± 3.5	18.2 ± 5.0	18.9 ± 4.7	0.46**^c^**

Bands,	Septic-CG	2.8 ± 4.1	38.1 ± 8.2*	28.6 ± 19.8	0.021**^a^**
%	Low-MAP	11.6 ± 15.6	57.2 ± 9.6	23.4 ± 18.2**^#^**	0.05**^b^**
	High-MAP	0.8 ± 0.7	63.9 ± 8.6	27.4 ± 10.0	0.59**^c^**

Metamyelocytes,	Septic-CG	0.0 ± 0.0	14.3 ± 12.1*	12.5 ± 7.5	0.001**^a^**
%	Low-MAP	0.0 ± 0.0	6.2 ± 3.3	1.9 ± 3.6**^#^**	0.93**^b^**
	High-MAP	0.0 ± 0.0	6.5 ± 4.1	1.4 ± 3.0	

Creatinine,	Septic-CG	0.5 (0.4, 0.8)	1.6 (1.4, 1.6)*	2.3 (2.0, 2.6)	<0.03**^d^**
mg/dl, median (IQR)	Low-MAP	0.9 (0.8, 1.0)	1.2 (1.0, 1.7)	1.4 (1.0, 2.1)**^#^**	0.295^e^
	High-MAP	0.9 (0.8, 0.9)	1.1 (0.9, 1.2)	0.9 (0.7, 1.0)	0.001**^f^**
					0.021^g^

Total bilirubin,	Septic-CG	1.6 ± 1.2	1.0 ± 0.0*	1.1 ± 0.4	>0.60**^d^**
µmol/L	Low-MAP	1.5 ± 1.1	1.4 ± 1.1	1.6 ± 1.5**^#^**	
	High-MAP	1.3 ± 0.5	1.1 ± 0.4	1.4 ± 0.7	

Creatine-kinase,	Septic-CG			475 (360, 779)	0.045**^f^**
U/L, median (IQR)	Low-MAP			253 (232, 470)	0.28**^g^**
	High-MAP			211 (183, 332)	

Troponin,	Septic-CG			0.015 (0.013, 0.032)	0.091**^f^**
µg/L, median (IQR)	Low-MAP			0.008 (0.05, 0.028)**a^#^**	
	High-MAP			0.007 (0.006, 0.02)	

Values are mean ± SD or median (IQR) where indicated. BL, baseline; EOP, end of observation period for Low-mean arterial pressure (MAP) and High-MAP groups (12 h after induction of peritonitis); END, end of the experiment (at 48 hours of resuscitation, or before death if earlier); ^#^data available for seven animals; *data available for five animals, in one measured after 24 h of PI, and in four after 18 h of PI; ^¶^*P*-value for ^a^time-group interaction with repeated measures analysis of variance (ANOVA) including BL, EOP and END for all study groups (septic control (Septic-CG), Low-MAP and High-MAP groups); ^b^time-group interaction with repeated measures ANOVA including BL, EOP and END for Low-MAP and High-MAP groups; ^c^independent samples *t*-test between Low-MAP and High-MAP groups at the END; ^d^time effect within each group (Septic-CG, Low-MAP and High-MAP) separately assessed by the non-parametric Friedman's test and including BL, EOP and END; ^e^Kruskal-Wallis test at the EOP; ^f^Kruskal-Wallis test at the End; ^g^Mann-Whitney *U*-test between Low-MAP and High-MAP groups at the END.

### Mitochondrial function

### Isolated skeletal muscle mitochondrial function

**Figure 3:**
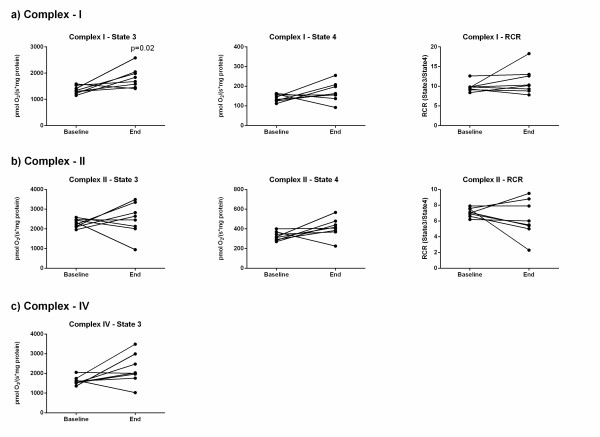
**Isolated skeletal muscle mitochondrial respiration during unresuscitated sepsis**. Complex I-, II- and IV-isolated skeletal muscle mitochondrial respiration in septic control animals from baseline to end (represented by connecting lines for each pig, n = 8). State 3 and State 4 oxygen consumption is expressed as pmol/second/mg protein. State 3, active respiration after addition of ADP; State 4, respiration after consumption of ADP. The ratio between State 3 and State 4 was calculated as the respiratory control ratio (RCR; state 3/state 4). *P = 0.02, paired *t*-test.

In the septic-CG, maximal skeletal muscle mitochondrial respiration (complex I-state 3) increased (baseline mean ± SD 1357 ± 149 pmol/s/mg; end 1822 ± 385 pmol/s/mg, paired *t*-test: *P *= 0.020), whereas complex I-state 4 and respiratory control ratio (RCR) (Figure 3a), and complex II- and IV-dependent skeletal muscle mitochondrial respiration did not change (Figure 3b, c). There were no changes over time or differences between the low- and high-MAP groups in the respiration of isolated muscle mitochondria (Figure S3 in Additional file 2).

### Isolated liver mitochondrial function

**Figure 4:**
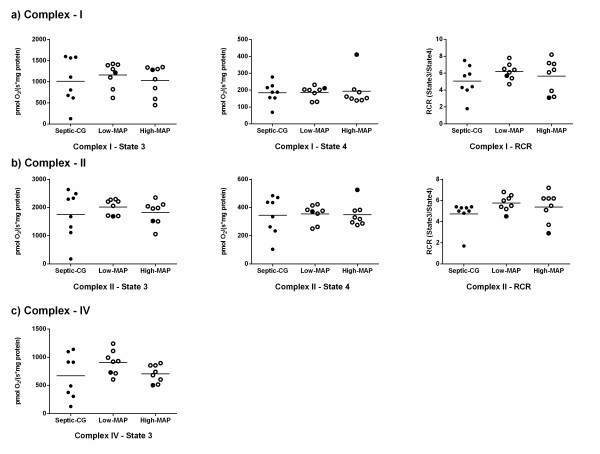
**Isolated liver mitochondrial respiration**. Comparison of isolated liver mitochondrial respiration in septic control animals and septic animals resuscitated to low-mean arterial pressure (MAP) and high-MAP targets (n = 8). State 3 and 4 oxygen consumption is expressed as pmol/second/mg protein. No significant differences were found between the groups (*P *>0.05, one-way analysis of variance). Horizontal lines represent mean values. Filled circles represent animals that died early. State 3, active respiration after addition of ADP; State 4, respiration after consumption of ADP; RCR, respiratory control ratio (oxygen consumption of State 3/State 4).

No significant differences between groups were observed for complex I-, II- or IV-dependent respirations of isolated liver mitochondria (Figure 4).

### Permeabilized skeletal muscle fibers mitochondrial function

Permeabilized skeletal muscle fibers complex I- and II-dependent respiration did not change in the low- and high-MAP groups (Table S3 in Additional file 2). However, complex IV-state 3 respiration increased in the low- and high-MAP groups during the resuscitation period (*P *= 0.003 for time effect) with no difference between the groups (Table S3 in Additional file 2). Permeabilized skeletal muscle fibers respiration did not change in the septic-CG (Table S4 in Additional file 2).

### Skeletal muscle ATP content

**Figure 5:**
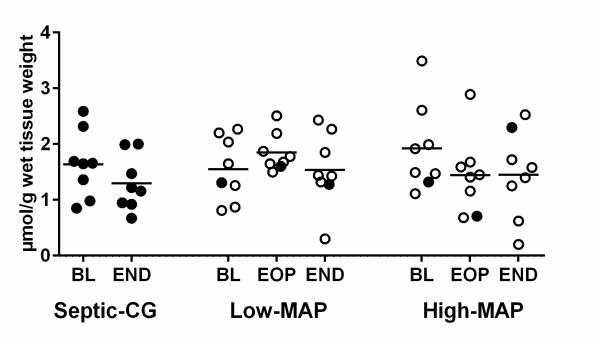
**Skeletal muscle tissue ATP concentrations in septic controls and animals resuscitated to low-mean arterial pressure (MAP) and high-MAP targets**. The black horizontal bars represent the mean values. Filled circles represent animals that died early. *P *>0.05 for all comparisons. BL, baseline; EOP, end of observation period; END, end of the experiment (at 48 h of resuscitation, or before death if earlier).

Skeletal muscle ATP content was preserved in all groups (Figure 5).

## Discussion

Blood pressure targets did not modify mitochondrial respiration or markers of inflammation. However, animals in the low-MAP group had lower SvO_2 _and hemoglobin levels, and a higher incidence of AKI, while animals in the high-MAP group had increased net positive fluid balance and vasopressor load during resuscitation. If the animals were not resuscitated after 12 h they died, with increased plasma levels of inflammatory markers, increased maximal skeletal muscle complex I mitochondrial respiration, and with preserved skeletal muscle ATP content.

Few clinical studies have prospectively addressed the effect on disease severity and the development of sepsis-associated organ dysfunction of different levels of MAP during the initial phase of severe sepsis and septic shock resuscitation [3-6]. The lack of available data supporting a specific MAP target during the initial resuscitation of septic patients is emphasized by the wide range of MAP goals (60 to 100 mmHg) applied in randomized controlled clinical trials [16]. Furthermore, experimental studies in sepsis either do not include MAP targets in resuscitation algorithms [22-24], or set the MAP target at 65 mmHg [25,26], consistent with the current recommendation of the Surviving Sepsis Campaign [2]. Nevertheless, no clinical or experimental studies have prospectively addressed the effects of lower MAP targets (50 to 60 mmHg) on outcomes.

Jhanji *et al*. have shown that in a case series of sixteen patients with septic shock, increasing MAP from 60 to 90 mmHg using norepinephrine improved systemic oxygen delivery and cutaneous PtO_2_, did not affect sublingual microvascular flow and did not improve urinary output [5]. In our study, systemic oxygen delivery increased similarly in the low- and high-MAP groups, and systemic oxygen consumption was maintained in all groups. However, four out of seven animals (57%) allocated to the low-MAP group, in comparison to none in the high-MAP group, presented with AKI at the end of study. In an experimental sepsis model using either endotoxemia or fecal peritonitis for 22 h with MAP >70 mmHg, Benes *et al*. demonstrated a similar overall incidence of AKI of 50% [27]. It has been postulated that higher MAP levels (approximately 70 to 80 mmHg) may be necessary to decrease the development of AKI in patients with septic shock [17]. In our study, animals allocated to attainment of MAP between 75 to 85 mmHg received higher amounts of both fluids and norepinephrine in comparison to animals allocated to the low-MAP group. We found a significant correlation between percentage of time spent with blood pressure >60 mmHg and plasma creatinine in the end of the study, but not between the amount of administered fluids and plasma creatinine. It seems unlikely therefore, that more fluid per se would have been beneficial in the low-MAP group.

Data from experimental [24,28] and clinical studies [15,29-37] have shown that therapeutic attempts, including administration of fluids and vasopressors to restore hemodynamic stability during sepsis may be associated with poor outcomes. In critically ill septic and non-septic patients, the cumulative positive fluid balance has been associated with a worse outcome [29-31,34,36,37], and the administration of catecholamines, particularly at higher doses, was associated with derangements in regional blood flow [7,8], adverse side effects [33,35] and increased likelihood of death [15,32,35]. In our experimental model such effects were not observed or observable. However, whether renal hyperfiltration as a result of high blood pressure and volume load may cause delayed harm to septic kidneys should be addressed in further studies.

Sepsis may result in various changes in mitochondrial enzyme activity and tissue ATP. Muscle complex-I activity decreased and complex-IV activity have been found to be increased in patients who subsequently died in sepsis [13], whereas an acute endotoxin challenge is reported to increase muscle complex-I activity in healthy volunteers [38]. Reduced tissue ATP levels have been observed in various tissues and sepsis models, but not consistently. Quadriceps muscle ATP was found to be reduced in non-survivors of sepsis but not in survivors [13]. In severe sepsis, intercostal muscle ATP is reported as normal, while leg muscle ATP is decreased [39]. In experimental sepsis decreased ATP content has been reported in the rat liver and the kidney but not in the muscle [40,41], and in the liver in porcine bacterial sepsis [42]. In porcine endotoxemia, reduced ATP content in the intestinal mucosa but not muscularis [43], as well as unchanged intestinal wall ATP has been reported [44].

Instead of respiratory chain enzyme activities, we assessed mitochondrial respiration using substrate-induced maximal oxygen consumption. We found no signs of impaired mitochondrial respiration in the muscle-isolated mitochondria or in the permeabilized muscle fibers, and the muscle ATP levels remained unchanged, even in the untreated septic group, with 100 % mortality. In contrast, complex I-dependent state 3 respiration of isolated muscle mitochondria increased in untreated sepsis, and complex IV-dependent state 3 respiration increased in the permeabilized muscle fibers in the resuscitated septic groups. In the presence of unchanged RCR and muscle ATP content, this may be related to increased metabolic activity and increased ADP transport into mitochondria [45]. One mechanism which may help to maintain ATP levels in sepsis is increased phosphocreatine breakdown, which provides fuel for various cellular processes [41].

We observed previously that delay in starting resuscitation was associated with decreased muscle ATP levels at the time of death [28], in contrast to the well-preserved muscle ATP levels in the present study. Deaths in that study occurred later, after a period of resuscitation, whereas in the present study the deaths occurred earlier, and all except two resuscitated animals survived. The well-preserved muscle ATP levels in the dying animals in the present study are likely to be related to the earlier death.

Resuscitation to a low compared to a high MAP target had no effect on isolated skeletal muscle mitochondrial respiration, or on isolated liver mitochondrial respiration, suggesting that neither higher organ perfusion pressure nor increased vasopressor load had an effect. We previously observed that the need to support hemodynamics with norepinephrine in porcine fecal peritonitis of shorter duration was associated with lower hepatic complex II-dependent state 4 respiration, while state 3 and RCR were not altered [20]. We also found in endotoxemic pigs that norepinephrine improved hepatic mitochondrial respiratory efficiency [46]. Similarly, norepinephrine tended to increase ileal wall mitochondrial complex-I activity in an endotoxemic sheep model [47]. The lack of difference in the liver mitochondrial respiration between the groups in the present study suggests that the effect of norepinephrine on the liver is model-dependent or related to the duration of exposure.

Our study has limitations. We evaluated only two ranges of MAP for the initial resuscitation of severe sepsis and septic shock. Second, respiration rates were not normalized to the citrate synthase activity (mitochondrial content) [48]. Reduced skeletal muscle mitochondrial enzyme activity has been observed in patients with sepsis and multiple organ failure if expressed per wet muscle weight, but not if normalized to citrate synthase activity, a marker of mitochondrial content [39]. This may suggest decreased mitochondrial mass rather than specific inhibition of the mitochondrial enzymes in sepsis. Consequently, our finding of increased permeabilized muscle fibers complex IV-respiration may be due to variations in mitochondrial content. Third, the final skeletal muscle and liver samples in the animals that died before completing the 48-hour resuscitation period were taken when MAP approached 30 mmHg, and therefore may potentially reflect a near-death state rather than non-confounded differences between the study groups. Fourth, we did not evaluate the microcirculation in animals resuscitated with a low MAP target. It is possible that lower MAP is associated with decreased microvascular blood flow [49]. Fifth, we studied a small number of animals per group. Therefore, there is a risk of a type II error, and we cannot exclude true, small, undetected differences between groups. Finally, in this model of fecal peritonitis in young, healthy pigs, MAP thresholds may not have the same importance for tissue oxygenation as in older septic patients.

## Conclusions

In this abdominal sepsis model with 100% mortality unless treatment is installed, the MAP targets of 50 to 60 mmHg versus 75 to 85 mmHg during resuscitation did not result in differences in the inflammatory response skeletal muscle ATP content, or in mitochondrial respiration. While targeting a lower MAP was associated with increased incidence of AKI, targeting a higher MAP resulted in increased net positive fluid balance and vasopressor load during resuscitation. The long-term effects on kidney function of using lower MAP targets, and whether the resulting increased fluid balance and vasopressor load using higher MAP targets are of relevance for recovery after initial resuscitation, need to be evaluated in further studies.

## Key messages

• Targeting a MAP between 50 and 60 mmHg in septic shock was associated with increased incidence of AKI.

• Targeting a higher MAP (75 to 85 mmHg) during resuscitation did not ameliorate the inflammatory response and resulted in increased net positive fluid balance and vasopressor load during resuscitation.

• MAP targets did not modify the inflammatory response, the skeletal muscle ATP content or mitochondrial respiration.

• Unresuscitated septic animals died, with increased plasma levels of inflammatory markers, increased maximal skeletal muscle complex-I mitochondrial respiration, and with preserved skeletal muscle ATP content.

## Abbreviations

AKI: acute kidney injury; ANOVA: analysis of variance; BL: baseline; CBFI: carotid artery flow index; CVP: central venous pressure; DO_2_: systemic oxygen delivery; EOP: end of observation period (12 hours after peritonitis induction); FBFI: femoral artery flow index; FiO_2_: fractional inspiratory oxygen concentration; G50%: glucose 50%; high-MAP: resuscitation targeting mean arterial pressure between 75 and 85 mmHg; IL-6: plasma interleukin-6; low-MAP: resuscitation targeting mean arterial pressure between 50 and 60 mmHg; MAP: mean arterial blood pressure; MPAP: mean pulmonary artery pressure; PaCO_2_: arterial carbon dioxide partial pressure; PAOP: pulmonary artery occlusion pressure; PEEP: positive end-expiratory pressure; RCR: respiratory control ratio; RP: resuscitation period; septic-CG: septic control group; SvO_2_: mixed venous oxygen saturation; SVRI: systemic vascular resistance index; TNF-α: tumor necrosis factor-α; VO_2_: systemic oxygen consumption.

## Competing interests

The authors declare that they have no competing interests.

## Authors' contributions

TDC, JT and SMJ devised the study protocol. TDC, MV, SD and SMJ initiated and performed all animal experiments. MV and SD performed mitochondria-related experiments. TDC, MV and SMJ analyzed the data. TDC and MV wrote the first manuscript draft. SMJ, JT and ES critically revised the manuscript. All authors approved the final manuscript.

## Supplementary Material

Additional file 1 **Word text file containing additional information about the methods, along with related references**. Click here for file

Additional file 2 **Word file containing four tables and three figures**. Table S1 indicates the percentage of time spent at different blood pressure ranges. Table S2 describes the acid-base-balance parameters and respiratory system variables. Table S3 gives permeabilized skeletal muscle fibers maximal mitochondrial respiration (state 3) in animals resuscitated to low and high-mean arterial pressure (MAP) targets. Table S4 lists permeabilized skeletal muscle fibers maximal mitochondrial respiration (state 3) in the septic control group. Figure S1 shows the MAP and norepinephrine administration over the study period in animals randomized to the low and high-MAP groups. Figure S2 shows a scatter plot of plasma creatinine in the three study groups. Figure S3 shows isolated skeletal muscle mitochondrial respiration in animals resuscitated to low and high MAP. Click here for file
