# Supplementary material for: Increasing mean arterial blood pressure in sepsis: effects on fluid balance, vasopressor load and renal function
Source: Crit Care. 2013 Jan 30;17(1):R21. doi: 10.1186/cc12495 (PMC4056362; doi:10.1186/cc12495)
Supplement: Additional file 1 — Word text file containing additional information about the methods, along with related references. [file cc12495-S1.DOCX]

**INCREASING MEAN ARTERIAL BLOOD PRESSURE IN SEPSIS: EFFECTS ON FLUID BALANCE, VASOPRESSOR LOAD AND RENAL FUNCTION**

**Additional file 1**

**Authors:**

Thiago Domingos Corrêa, Madhusudanarao Vuda, Jukka Takala, Siamak Djafarzadeh, Eliézer Silva and Stephan Mathias Jakob.

**Materials and methods**

*Hemodynamic support* *for Low-MAP and High-MAP groups*

Throughout the resuscitation period, the volume status was evaluated clinically every hour. If signs of hypovolemia became evident, alternating boluses of 150 mL Ringer’s lactate (RL) and 6% hydroxyethyl starch (HES 130/0.4) were given (Figure S1). Fluid boluses were repeated as long as the stroke volume was increased by 10% or more after fluid administration. The maximum dose of HES administered was 30 ml/kg. After this maximal dose was reached, only boluses of Ringer’s lactate were given.

If the mixed venous oxygen saturation (SvO_2_) was less than 50%, dobutamine administration was started at a dose of 5.0 mg per hour. This dose was increased by 5.0 mg per hour every 30 minutes until the SvO_2_ was 50% or higher or until a maximal dose of 20 mg per hour was given. If the mean arterial pressure (MAP) was less than 50 mmHg in Low-MAP group and less than 75 mmHg in the High-MAP group, norepinephrine was administrated.

*Ventilatory support for Low-MAP and High-MAP groups*

The animals were ventilated in a volume-controlled mode with a positive end-expiratory pressure (PEEP) of 5 cm H_2_O, a fraction of inspired oxygen (FiO_2_) of 30%, and a tidal volume (VT) of 8 mL/kg (Servo-i; Maquet Critical Care, Solna, Sweden). The respiratory rate was adjusted (20 to 35 breaths/min) to maintain a partial pressure of arterial carbon dioxide (PaCO_2_) at 35 - 45 mmHg.

Since prolonged septic state is a typical risk factor for acute lung injury, tidal volume, PEEP and FiO_2_ were adjusted during the resuscitation period following a standardized protocol with the aim of keeping the arterial oxygen partial pressure (PaO_2_) level between 100 and 150 mm Hg, the arterial oxyhemoglobin saturation (SpO_2_) (measured by pulse oximetry) level above 90%, and the plateau pressure below 30 cm H_2_O. All animals were intra-tracheally suctioned every 12 hours. When there was a sustained (> 10 minutes) decrease in oxygenation (SpO_2_ < 90% and/or PaO_2_ < 100 mm Hg, if available), the following stepwise procedures were performed: tracheal suctioning, a recruitment maneuver, and setting of a new FiO_2_ or PEEP according to a standardized protocol.

*Tracheal suctioning*

Tracheal suctioning was carried out every 12 hours and when any one of the following situations occurred: visible airway secretion into the endotracheal tube, noisy breathing, increased inspiratory peak pressure, decreased VT during ventilation attributed to airway secretion, and decreased SpO_2_ during ventilation attributed to airway secretion.

Before aspirations, the animals were preoxygenated at 100% oxygen for 2 minutes. Aspirations were performed using a closed tracheal suctioning system, at a negative pressure, for 20 seconds, during which the catheter was gently rotated and withdrawn. After an aspiration procedure, a recruitment maneuver was performed if there were no contraindications.

*Lung recruitment maneuver*

A recruitment maneuver (RM) was performed as follows: FiO_2_ was increased to 100% for 2 minutes before a RM. The tidal volume was increased over 30 seconds to 10 ml/kg. Then, a 15-second inspiratory hold was performed. If there are no signs of hemodynamic instability, another inspiratory hold of 15-second is performed with an interval between the holds of 60 seconds. Afterwards, tidal volume was decreased over 30 seconds to the pre-RM level. No more than six recruitment maneuvers were performed in any 24-hour period.

The RM was terminated immediately and VT restored to its pre-RM level if there was any sign of hemodynamic instability and/or the mean arterial blood pressure decreased by 10 mmHg or more.

*Combination of PEEP and FiO_2_*

If arterial oxygenation was not within the target range after the two procedures described above, then either FiO_2_ or PEEP was adjusted according to Table S1. When arterial oxygenation was higher than the target range (PaO_2_ ≥ 150 mmHg), either the FiO_2_ or the PEEP was decreased (by 10% or 2.0 cm H_2_O, respectively), depending on which was farther (number of changes in value) from the beginning of the target scale shown in Table S1. If PEEP and FiO_2_ were equidistant from the scale, then FiO_2_ was decreased first. If arterial oxygenation was lower than the target range (PaO_2_ < 100 mmHg or SpO_2_ < 90%), either FiO_2_ or PEEP was increased (by 10% or 2.0 cm H_2_O, respectively), depending on which was farther from the end of the target scale. If PEEP and FiO_2_ were equidistant from the scale, then PEEP was increased first (Table S1). If FiO_2_ = 100%, PEEP was increased in 2 cm H_2_O increments up to 24 cm H_2_O.

*Blood glucose management*

Blood glucose (BG) concentration was maintained between 3.5 and 5.0 mmol/l throughout the study period following a protocolized algorithm (Figure S2). BG measurement was performed hourly or two-hourly using ACCU-CHEK glucometer (Roche Diagnostics, Mannheim, Germany).

*Management of blood glucose ≤ 3.5 mmol/l*: For all pigs, glucose 50% solution was started when BG was lower or equal to 3.5 mmol/L at rate of 0.2 ml/kg/h. Afterwards, if BG ≤ 3.5 mmol/l, G50% infusion was increased in 4 ml/h hourly, until BG concentration between 3.5 - 5.0 mmol/l. If BG concentration was between 3.5 - 5.0 mmol/l, the G50% infusion rate was kept unchanged. If BG > 5.0 mmol/l, G50% infusion rate was reduced by 2.0 ml/h hourly until BG concentration stayed between 3.5 - 5.0 mmol/l. When BG was not in the target range or after any adjustments on G50% infusion rate, BG measurements were perform hourly. After two measurements with BG in the target range, BG was measured two-hourly (Figure S2).

*Management of blood glucose > 5.0 mmol/l*: G50% infusion rate was reduced in 2 ml/h until stop G50% infusion. BG measurement was performed hourly. When BG ≤ 3.5 mmol/l, G50% infusion was restarted at 0.2 ml/kg/h (Figure S2).

*Blood sampling*

Blood from the carotid artery was withdrawn at baseline and every six hours from the indwelling catheter and immediately analyzed in a blood gas analyzer (GEM Premier 3000 analyzer; Bedford, MA, USA) for PaO_2_, PaCO_2_ (adjusted to central body temperature), pH, lactate (mmol/L), base excess (BE), sodium and potassium. Arterial oxygen saturation and total hemoglobin concentration (Hb) were measured at baseline and every six hours using a separate analyzer (OSM 3; Radiometer, Copenhagen, Denmark, porcine mode). Blood from the pulmonary artery was withdrawn at baseline and every six hours for oxygen saturation measurement using a Radiometer OSM 3 blood gas analyzer (OSM 3; Radiometer, Copenhagen, Denmark, porcine mode).

Blood samples were taken from the carotid artery for hemoglobin, platelets and white blood cell count (ADVIA^®^ 120 analyzer; Siemens, Zürich, Switzerland) at baseline, the end of the observation period and at the end of the study (48 hours of resuscitation or before death, for animals that died before completion of the study).

Plasma interleukin-6 (IL-6) and tumor necrosis factor alpha (TNF-alpha) were determined using a porcine immunoassay kit (R&D Systems Europe Ltd; Abingdon, Oxon, UK). IL-6, TNF-alpha, serum creatinine and total bilirubin were measured with blood samples from the carotid artery at baseline, the end of the observation period and at the end of the study (48 hours of resuscitation or before death, for animals that died before completion of the study). Creatin-Kinase and troponin were measured with blood samples from the carotid artery at the end of the study (48 hours of resuscitation or before death, for animals that died before completion of the study).

*Preparation of permeabilized skeletal muscle fibers*

Skeletal muscle fibers were isolated by dissection of the muscle tissue in relaxing solution (in mM: 2.77 CaK_2_EGTA, 7.23 K_2_EGTA [free Ca^2+^ concentration 0.1 µM], 20 imidazole, 20 taurine, 6.56 MgCl_2_, 5.77 ATP, 15 phosphocreatine, 0.5 dithiothreitol, and 50 K-MES, pH 7.1) on ice and permeabilized by gentle agitation for 30 min on ice in the relaxing solution supplemented with 50 µg/ml saponin [1]. Fibers were washed in ice-cold respiration medium (110 mM sucrose, 60 mM K-lactobionate, 0.5 mM EGTA, 1 g/L bovine serum albumin essentially fatty acid-free, 3 mM MgCl_2_, 20 mM taurine, 10 mM KH_2_PO_4_, 20 mM HEPES, pH 7.1) [2] by agitation for 20 min and were kept in this medium until respirometric assay.

*Permeabilized skeletal muscle fibers mitochondrial function analysis*

State 3 respiration was measured using exogenous substrates glutamate/malate (complex I activation), succinate (complex II activation) and artificial substrates ascorbate/TMPD (complex IV activation). In addition, maximal electron transport system capacity was also measured in the presence of substrates for both complex I and complex II.

*CALCULATIONS*

Systemic vascular resistance index (SVRI), systemic oxygen delivery (DO_2_) and systemic oxygen consumption (VO_2_) were calculated using the following formulas:

SVRI = (MAP-CVP)/CI;

DO_2_ = CO x CaO_2_;

VO_2_ = CO x (CaO_2_ - CvO_2_);

CaO_2_ = (Hb x SaO_2_ x 1.34);

CvO_2_ = (Hb x SvO_2_ x 1.34);

Where MAP = Mean arterial blood pressure, CVP = Central venous pressure, CI = Cardiac index, CO = Cardiac output, CaO_2_ = Arterial oxygen content, Hb = Hemoglobin, SaO_2_ = Arterial oxyhemoglobin saturation, SvO_2_ = Mixed venous oxygen saturation.

**References**

1. Kuznetsov AV, Mayboroda O, Kunz D, Winkler K, Schubert W, Kunz WS: **Functional imaging of mitochondria in saponin-permeabilized mice muscle fibers.** *J Cell Biol* 1998, **140:**1091-1099.

2. Gnaiger E, kuznetsov AV, Schneeberger S, Seiler R, Brandacher G, Steurer W, Margreiter R: **Mitochondria in the cold.** In *Life in the cold*. Edited by Heldmaier G, Klingenspor M. Springer-Verlag Berlin Heidelberg New York; 2000:431-442.

**Figure S1:** Hemodynamic protocol.


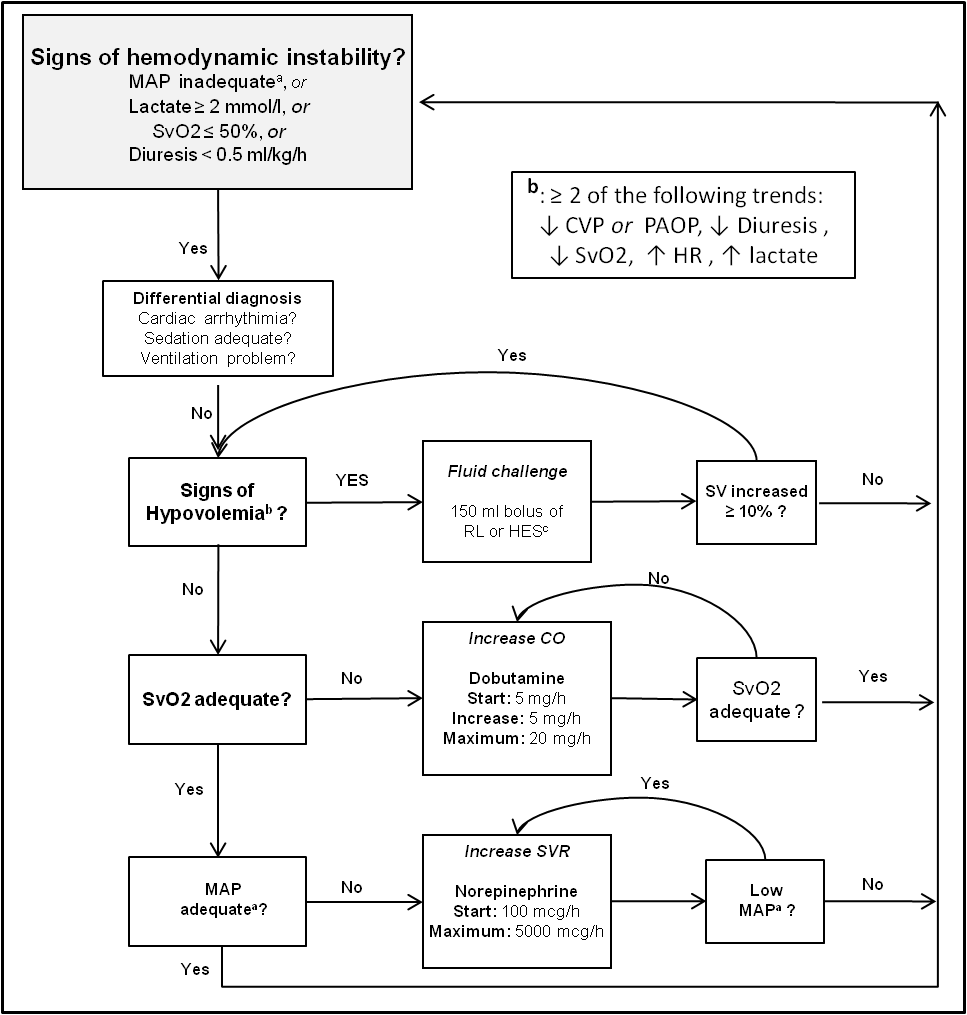


Legend: MAP, mean arterial blood pressure; SvO_2_, mixed venous oxygen saturation; SV, stroke volume; CVP, central venous pressure; PAOP, pulmonary artery occlusion pressure; SVR, systemic vascular resistance; RL, Ringer’s lactate; HES, 6% hydroxyethyl starch (130/0.4); ↑, increasing; ↓, decreasing; a, targeting MAP between 50-60 mmHg for Low-MAP group and between 75-85 mmHg for High-MAP group; c, alternating boluses of 150 mL Ringer’s lactate and 6% hydroxyethyl starch (HES 130/0.4) until the maximum dose of 30 ml/Kg of HES is reached. Then, only give boluses of Ringer’s lactate.

**Figure S2:** Blood glucose management protocol.


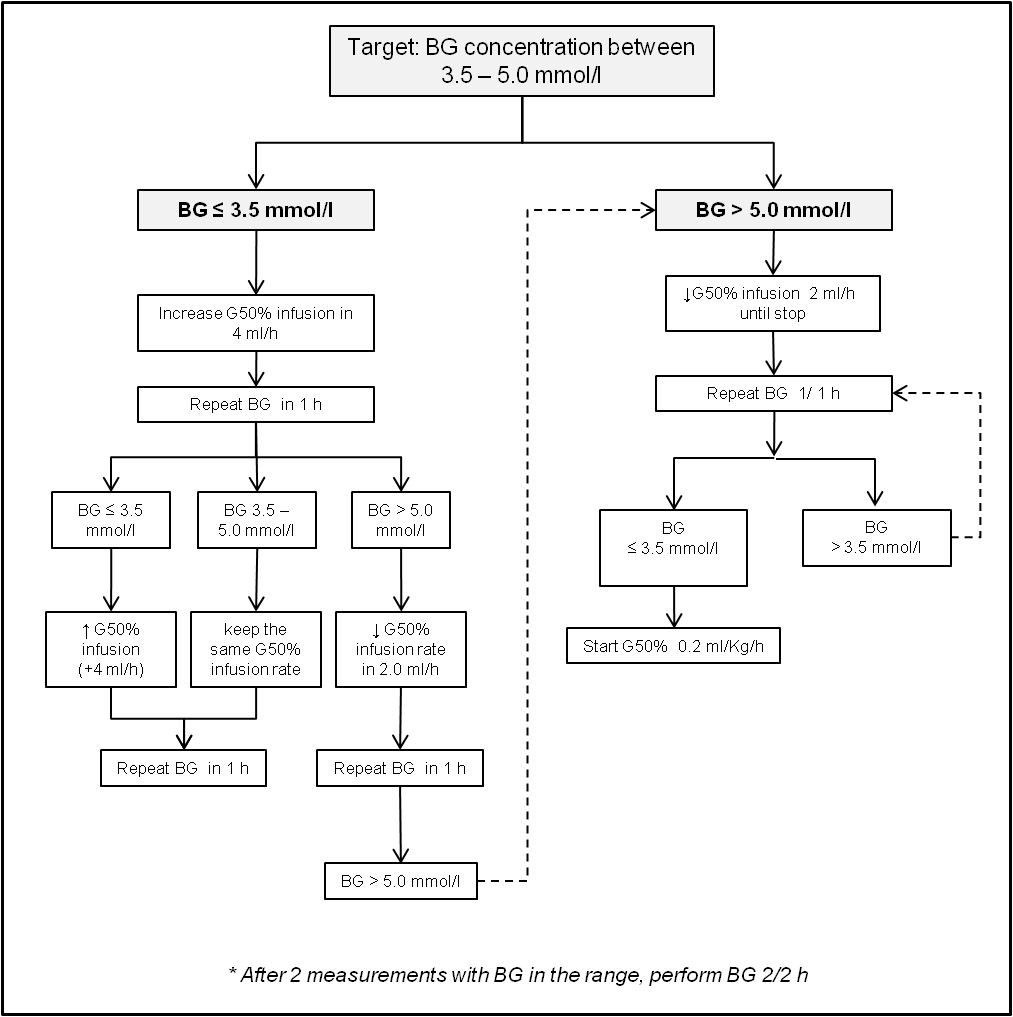


Legend: BG, blood glucose; G50%, glucose 50% solution; ↑, increase; ↓, decrease.

**Table S1.** Combinations of PEEP and FiO_2_.

| **FiO_2_** (%) | 0.4 | 0.5 | 0.6 | 0.7 | 0.8 | 0.9 | 1.0 |
| --- | --- | --- | --- | --- | --- | --- | --- |
| **PEEP** (cm H_2_O) | 6 | 8 | 10 | 12 | 14 | 16 | 18-24 |

Legend: FiO_2_, fraction of inspired oxygen and PEEP, positive end expiratory pressure.
