# Supplementary material for: Increasing mean arterial blood pressure in sepsis: effects on fluid balance, vasopressor load and renal function
Source: Crit Care. 2013 Jan 30;17(1):R21. doi: 10.1186/cc12495 (PMC4056362; doi:10.1186/cc12495)
Supplement: Additional file 2 — Word file containing four tables and three figures. Table S1 indicates the percentage of time spent at different blood pressure ranges. Table S2 describes the acid-base-balance parameters and respiratory system variables. Table S3 gives permeabilized skeletal muscle fibers maximal mitochondrial respiration (state 3) in animals resuscitated to low and high-mean arterial pressure (MAP) targets. Table S4 lists permeabilized skeletal muscle fibers maximal mitochondrial respiration (state 3) in the septic control group. Figure S1 shows the MAP and norepinephrine administration over the study period in animals randomized to the low and high-MAP groups. Figure S2 shows a scatter plot of plasma creatinine in the three study groups. Figure S3 shows isolated skeletal muscle mitochondrial respiration in animals resuscitated to low and high MAP. [file cc12495-S2.DOCX]

**INCREASING MEAN ARTERIAL BLOOD PRESSURE IN SEPSIS: EFFECTS ON FLUID BALANCE, VASOPRESSOR LOAD AND RENAL FUNCTION**

**Additional file 2**

**Authors:**

Thiago Domingos Corrêa, Madhusudanarao Vuda, Jukka Takala, Siamak Djafarzadeh, Eliézer Silva and Stephan Mathias Jakob.

**Table S1.** Percentage of time at different mean arterial blood pressure (MAP) ranges during the resuscitation period.

| **Low-MAP Group** |  |  |
| --- | --- | --- |
| MAP levels | % time | Cumulative % |
| < 50 mmHg | 2.3 | 2.3 |
| 50 - 60 mmHg | 28.8 | 31.1 |
| 61 - 74 mmHg | 41.4 | 72.5 |
| > = 75 mmHg | 27.5 | 100.0 |
|  |  |  |
| **High-MAP Group** |  |  |
| MAP levels | % time | Cumulative % |
| < = 60 mmHg | 0.5 | 0.5 |
| 61 - 74 mmHg | 7.6 | 8.1 |
| 75 - 85 mmHg | 54.4 | 62.5 |
| > 85 mmHg | 37.5 | 100.0 |

**Table S2.** Arterial blood gases and respiratory system variables. Values are mean ± SD.

| **Variables** | **Group** | **BL** | **EOP** | **RP 12h** | **RP 24h** | **RP 36h** | **RP 48h** | **END** | **p value ^¶^** |
| --- | --- | --- | --- | --- | --- | --- | --- | --- | --- |
| pH | Septic-CG | 7.54 ± 0.05 | 7.41 ± 0.06 |  |  |  |  | 7.28 ± 0.05 | 0.002 ^a^ |
|  | Low-MAP | 7.50 ± 0.03 | 7.43 ± 0.04 | 7.44 ± 0.05 | 7.44 ± 0.09 | 7.45 ± 0.06 | 7.44 ± 0.07 | 7.41 ± 0.12 | 0.93 ^b^ |
|  | High-MAP | 7.52 ± 0.04 | 7.45 ± 0.03 | 7.49 ± 0.03 | 7.49 ± 0.06 | 7.49 ± 0.04 | 7.46 ± 0.05 | 7.44 ± 0.07 |  |
| Bicarbonate | Septic-CG | 32.4 ±1.4 | 26.7 ± 3.2 |  |  |  |  | 19.8 ± 2.4 | <0.001 ^a^ |
| (mmol/L) | Low-MAP | 30.4 ± 2.2 | 27.0 ± 1.0 | 27.5 ± 1.9 | 29.9 ± 2.5 | 30.2 ± 3.4 | 30.6 ± 3.1 | 30.3 ± 3.0 | 0.43 ^b^ |
|  | High-MAP | 31.9 ± 1.4 | 27.5 ± 1.1 | 28.9 ± 1.9 | 30.0 ± 2.5 | 30.3 ± 1.5 | 30.8 ± 1.4 | 29.8 ± 3.0 |  |
| BE | Septic-CG | 9.7 ± 1.7 | 3.1 ± 3.6 |  |  |  |  | -5.6 ± 2.7 | <0.001 ^a^ |
| (mmol/L) | Low-MAP | 7.7 ± 1.7 | 3.7 ± 1.4 | 4.2 ± 2.0 | 6.3 ± 3.2 | 6.5 ± 3.6 | 6.7 ± 3.7 | 5.8 ± 4.2 | 0.61 ^b^ |
|  | High-MAP | 9.3 ± 1.7 | 4.5 ± 1.4 | 6.4 ± 2.2 | 7.2 ± 3.0 | 7.3 ± 1.7 | 7.1 ± 1.0 | 6.0 ± 3.3 |  |
| PaCO_2_ | Septic-CG | 39.0 ± 4.1 | 42.9 ± 3.2 |  |  |  |  | 44.4 ± 5.3 | 0.471 ^a^ |
| (mm Hg) | Low-MAP | 40.3 ± 4.3 | 42.1 ± 2.7 | 41.9 ± 7.0 | 45.8 ± 10.7 | 45.1 ± 5.5 | 46.4 ± 5.3 | 50.8 ± 13.2 | 0.86 ^b^ |
|  | High-MAP | 39.6 ± 3.5 | 40.8 ± 2.7 | 38.3 ± 1.1 | 40.4 ± 3.6 | 41.0 ± 3.5 | 45.1 ± 6.2 | 45.4 ± 5.8 |  |
| MV | Septic-CG | 7.0 ± 0.3 | 7.5 ± 1.0 |  |  |  |  | 8.0 ± 0.9 | 0.001 ^a^ |
| (L/min) | Low-MAP | 7.5 ± 0.8 | 7.8 ± 0.7 | 8.3 ± 1.0 | 9.3 ± 1.8 | 10.8 ± 1.7 | 10.9 ± 2.0 | 10.7 ± 1.9 | 0.08 ^b^ |
|  | High-MAP | 7.0 ± 0.6 | 8.0 ± 1.0 | 8.6 ± 1.2 | 8.8 ± 0.9 | 8.9 ± 1.2 | 9.9 ± 0.9 | 9.8 ± 0.9 |  |
| PaO_2_/FiO_2_ | Septic-CG | 435 ± 48 | 402 ± 45 |  |  |  |  | 375 ± 49 | <0.001 ^a^ |
|  | Low-MAP | 451 ± 24 | 393 ± 50 | 351 ± 106 | 255 ± 95 | 210 ± 55 | 216 ± 68 | 197 ± 83 | 0.20 ^b^ |
|  | High-MAP | 455 ± 21 | 395 ± 41 | 325 ± 59 | 234 ± 36 | 223 ± 50 | 183 ± 74 | 169 ± 80 |  |
| PEEP | Septic-CG | 5 ± 0 | 5 ± 0 |  |  |  |  | 5 ± 0 | <0.001 ^a^ |
| (cm H_2_0) | Low-MAP | 5 ± 0 | 5 ± 0 | 6 ± 2 | 7 ± 4 | 8 ± 3 | 10 ± 2 | 11 ± 3 | 0.29 ^b^ |
|  | High-MAP | 5 ± 0 | 5 ± 0 | 5 ± 1 | 8 ± 2 | 9 ± 2 | 12 ± 4 | 12 ± 4 |  |

Legend: BL, baseline; EOP, 12 hours after peritonitis induction (End of observation period for Low MAP and High MAP groups); RP, resuscitation period; END, end of the experiment (at 48 hours of resuscitation or before death if earlier); pH, arterial pH; BE, arterial base excess; PaCO_2,_ arterial carbon dioxide partial pressure; MV, expired minute volume; PaO_2_/FiO_2_, arterial oxygen partial pressure and fraction of inspired oxygen relationship; PEEP, positive end-expiratory pressure; ¶, p value: a, time-group interaction with repeated measures ANOVA including BL, EOP and END for all study groups (Septic-CG, Low-MAP and High-MAP); b, time-group interaction with repeated measures ANOVA including BL, EOP, RP 12h, RP 24h, RP 36h and RP 48h for Low-MAP and High-MAP groups.

**Table S3:** Permeabilized skeletal muscle fibers maximal mitochondrial respiration (state 3) in animals resuscitated to low and high-MAP targets. Data expressed as pmole/(sec* mg dry weight).

| State 3 | Group | BL | EOP | End | P value ^¶^ | Paired t-test^*^ |
| --- | --- | --- | --- | --- | --- | --- |
| Complex I | Low-MAP | 137 ± 52 | 110 ± 19 | 136 ± 29 | 0.452 ^a^  0.452 ^b^  0.592 ^c^ |  |
|  | High-MAP | 115 ± 52 | 119 ± 51 | 124 ± 37 |  |  |
| Complex I + II | Low-MAP | 165 ± 63 | 129 ± 28 | 175 ± 54 | 0.398 ^a^  0.323 ^b^  0.846 ^c^ |  |
| (ETS Capacity) | High-MAP | 159 ± 49^#^ | 153 ± 63 | 173 ± 51 |  |  |
| Complex II | Low-MAP | 122 ± 50 | 100 ± 22 | 135 ± 51 | 0.605 ^a^  0.339 ^b^  0.591 ^c^ |  |
|  | High-MAP | 128 ± 44^#^ | 113 ± 40 | 121 ± 29 |  |  |
| Complex IV | Low-MAP | 76 ± 31 | 59 ± 14 | 99 ± 48 | 0.862 ^a^  0.003 ^b^  0.465 ^c^ | BL vs. EOP: p=0.133 |
|  | High-MAP | 78 ± 22 | 72 ± 28 | 107 ± 28 |  | EOP vs. End: p=0.001 |

Legend: values are mean ± SD. BL, baseline; EOP, end of observation period; ¶, p value: a, time-group interaction with repeated measures ANOVA; b, time effect with repeated measures ANOVA; c, group effect with repeated measures ANOVA; #, Data available for 7 animals; *, paired t-test: since there was no time-group interaction, paired t-tests were conducted on the pooled data from all groups and BL was compared to the EOP and EOP to the End.

**Table S4:** Permeabilized skeletal muscle fibers maximal mitochondrial respiration (state 3) in septic control group. Data expressed as pmole/(sec* mg dry weight). Values are mean ± SD.

| State 3 | Baseline | End | P value ^¶^ |
| --- | --- | --- | --- |
| Complex I | 130 ± 68 | 139 ± 98 | 0.845 |
| Complex I +II  (ETS Capacity) | 187 ± 103 | 190 ± 135 | 0.962 |
| Complex II | 137 ± 60 | 139 ± 99 | 0.944 |
| Complex IV | 99 ± 49 | 101 ± 76 | 0.944 |

Legend: ¶, p value for paired t-test.

**Figure S1:** Mean arterial blood pressure (MAP) and norepinephrine administration over the study period.

**

**

Legend: Legend: BL, baseline; EOP, end of observation period; END, end of the experiment (at 48 hours of resuscitation or before death if earlier); dotted black lines, animals randomized to Low and High-MAP groups who died during the resuscitation period; white filled circles, the beginning of norepinephrine administration and black filled circles, the time when norepinephrine was stopped.

**Figure S2:** Scatter plot of plasma creatinine of three study groups.





Legend: BL, baseline; EOP, 12 hours after peritonitis induction (End of observation period for Low-MAP and High-MAP groups); END, end of the experiment (at 48 hours of resuscitation or before death if earlier); #, data available for 7 animals; *, data available for 5 animals; a, time effect within each group (Septic-CG, Low-MAP and High-MAP) separately accessed by the non-parametric Friedman’s test including BL, EOP and END; b, Kruskal-Wallis test at the EOP; c, Kruskal-Wallis test at the End; d, Mann-Whitney U test between Low-MAP and High-MAP groups at the END. Horizontal lines represent median values. Filled circles represent animals that died early.

Figure S3: Isolated skeletal muscle mitochondrial respiration in animals resuscitated to Low and high MAP.


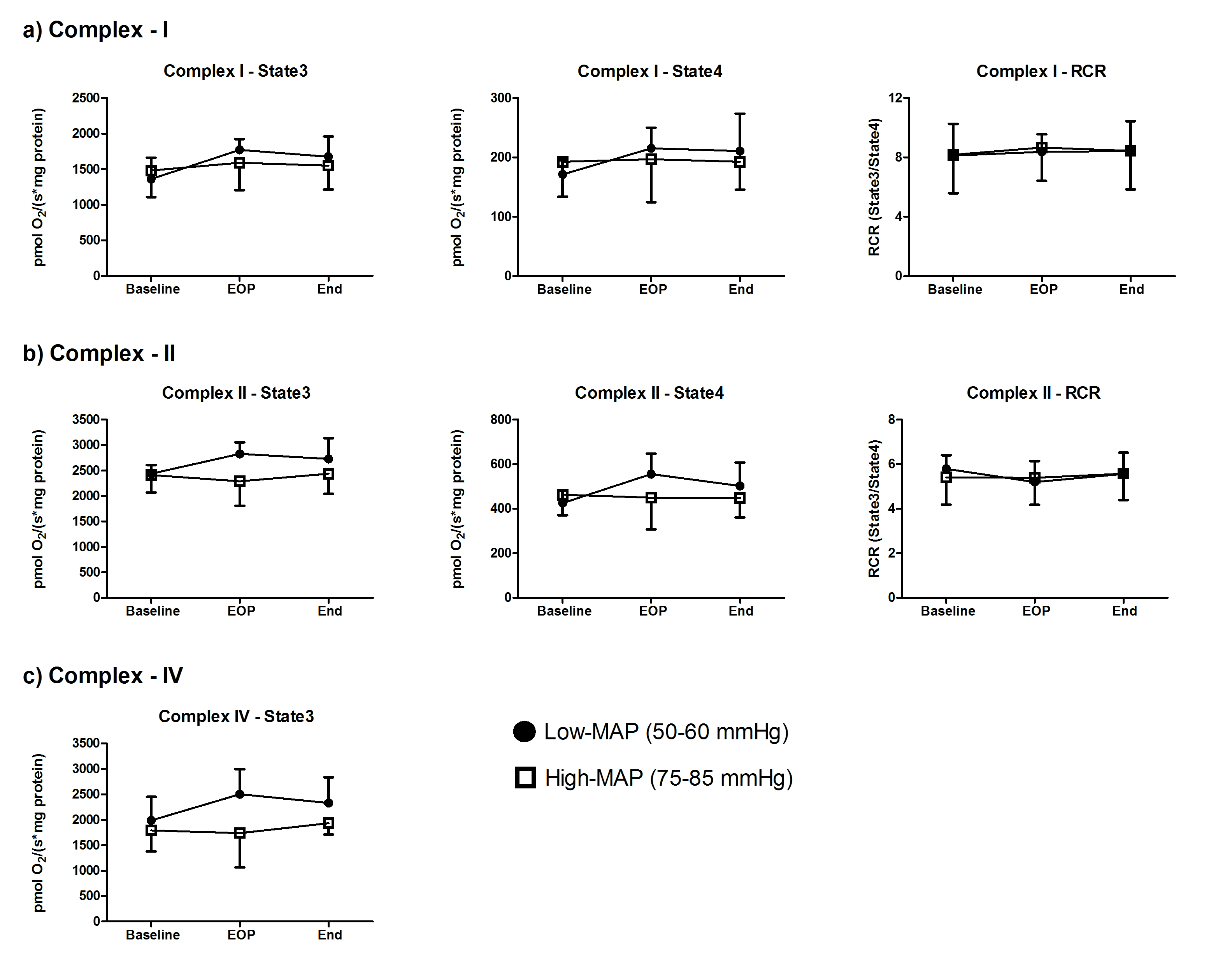


Legend: Complex I-, II- and IV-dependent isolated skeletal muscle mitochondrial respiration from septic animals resuscitated to Low-MAP and High-MAP targets. EOP, end of observation period. State 3 and state 4 oxygen consumption is expressed as pmol/s/mg protein. State 3, active respiration after addition of ADP. State 4, respiration after consumption of ADP. The ratio between state 3 and state 4 was calculated as the respiratory control ratio (RCR; state 3/state 4). Data represents mean ± SD (n=8).
